# Immunocompetent cells in durable ventricular assist device-implanted non-ischaemic dilated cardiomyopathy

**DOI:** 10.1007/s11748-022-01773-y

**Published:** 2022-02-28

**Authors:** Ayumi Koga-Ikuta, Satsuki Fukushima, Hatsue Ishibashi-Ueda, Naoki Tadokoro, Takashi Kakuta, Takurya Watanabe, Norihide Fukushima, Ken Suzuki, Toshihiro Fukui, Tomoyuki Fujita

**Affiliations:** 1 Department of Cardiac Surgery, National Cerebral and Cardiovascular Center, Osaka, Japan; 2 Department of Cardiovascular Surgery, Kumamoto University Graduate School of Medical Sciences, Kumamoto, Japan; 3 Department of Pathology, National Cerebral and Cardiovascular Center, Osaka, Japan; 4 Department of Transplantation, National Cerebral and Cardiovascular Center, Osaka, Japan; 5 Translational Therapeutics, William Harvey Research Institute, Barts and The London School of Medicine and Dentistry, Queen Mary University of London, London, UK

**Keywords:** Left ventricular assist device, Non-ischaemic dilated cardiomyopathy, Immunocompetent cells

## Abstract

**Objective:**

Because the presence of immunocompetent cells in the myocardium is associated with the pathological stage and/or myocardial viability, we explored relationships between functional recovery after left ventricular assist device implantation and the distribution of immunocompetent cells in non-ischaemic dilated cardiomyopathy patients.

**Methods:**

We reviewed 50 consecutive dilated cardiomyopathy patients implanted with HeartMate II at our institute between April 2013 and December 2018 who were treated with optimal medical therapy during left ventricular assist device support. Patients were stratified by improvement of the left ventricular ejection fraction at 6 months after implantation: ≥ 10% increase (Gr ≥ 10%), 5–10% (Gr 5–10%), and ≤ 5% (Gr ≤ 5%). T cells and macrophages were evaluated in the apical myocardium after left ventricular assist device implantation.

**Results:**

During left ventricular assist device support, 12 patients underwent heart transplantation and 2 patients died. Four patients with Gr ≤ 5% were readmitted because of congestive heart failure, but none with Gr ≥ 10%. Macrophages and T cells in the left ventricular myocardium with Gr ≥ 10% were significantly more present compared to those in other groups.

**Conclusions:**

The distribution of immunocompetent cells in the left ventricular myocardium might predict myocardial viability of this pathology after implantation.

## Introduction

Non-ischaemic dilated cardiomyopathy (NIDCM) is the most prominent pathology influenced clinically by the degree of functional recovery under durable left ventricular assist device (LVAD) support. NIDCM is generally a progressive pathology that potentially leads to abolition of left ventricular (LV) functions, which induces aortic insufficiency [1]. The major population affected by NIDCM is young adults who are more likely to present with congestive heart failure than older adults.

LVAD implantation reverses LV remodelling by unloading the LV [2]. Reduction of LV wall stress by LVAD implantation induces various cellular and molecular processes that consequently reverse LV remodelling [3]. It has been reported that the processes occasionally recover LV functions to the point where patients can be successfully weaned from LVAD [4, 5]. A potential crucial factor in this process is immunocompetent cells, such as T cells and macrophages, which regulate inflammation, fibrosis, and interstitial construction of the myocardium [6–8]. Immunocompetent cells are abundantly present in the myocardium and play various important roles in the heart [9]. Among them, it is well known that myocardial insults, such as myocardial infarction, activate cardiac macrophages and T cells to trigger innate inflammation that is subsequently suppressed to maintain the cardiac structure by fibrogenesis [10–12]. Additionally, the presence of immunocompetent cells in the myocardium is reportedly associated with the pathological stage and/or myocardial viability of NIDCM patients. However, the role of immunocompetent cells in the NIDCM heart under LVAD support is not fully understood. Furthermore, the relationships between functional recovery and immunocompetent cells in NIDCM are poorly understood. Here, we explored relationships between functional recovery post-LVAD implantation and the presence of immunocompetent cells in the myocardium of advanced NIDCM patients.

## Subjects

The institutional surgical database contained a consecutive series of 50 patients who underwent HeartMate II^®^ (Abbott, Chicago, IL, USA) implantation for advanced NIDCM at the National Cerebral and Cardiovascular Center between April 2013 and December 2018. Data were collected from medical charts, operation reports, and referral letters in December 2018.

## Methods

### Surgical indication and LVAD implantation procedure

Surgical indication of LVAD implantation was discussed by the institutional multidisciplinary heart team, which comprised cardiologists, surgeons, transplant physicians, transplant coordinators, and co-medical staff, who particularly focussed on medical and social indications of heart transplantation because an LVAD was implanted for “bridge-to-transplantation” purposes. Selection of the LVAD device was subsequently determined by (1) anatomies of the heart and adjacent organs/tissues, (2) the structure and functions of the heart, and 3) proficiency in device handling of the patient and family.

Implantation of the LVAD was performed via the median sternotomy approach. Additional tricuspid annuloplasty using a prosthetic ring or aortic valve plasty by a coaptation stitch [13] was performed at the time of LVAD implantation surgery in cases with mild or more tricuspid or aortic regurgitation, respectively. Tricuspid annuloplasty was performed under heart beating, whereas aortic valve plasty was performed under induced cardiac arrest.

### Post-operative treatment and follow-up

Post-operatively, all cases were treated at the intensive care unit until sufficient recovery for transfer to the general ward. Anti-coagulant therapy by continuous heparin infusion was started within 24 h post-operatively, while the heparin dose was titrated to maintain an activated partial thromboplastin time of 50–70 s. Subsequently, 100 mg aspirin per day and warfarin were administered to target INR 2.5–3.5 when oral intake was restarted. After stabilising patient haemodynamics, aggressive medical treatments by β-blockers, angiotensin-converting enzyme inhibitors, and/or spironolactone were started, which depended on blood pressure, heart rate, and renal functions. After discharge from the hospital, all cases regularly visited the institutional outpatient clinic for review.

### Laboratory data collection and analysis

All patients were examined by standard transthoracic echocardiography within 7 days prior to LVAD implantation and at 6 months after LVAD implantation. Standard data, such as LV end-diastolic dimension (LVDD), end-systolic dimension (LVSD), and LV ejection fraction (LVEF) of the LV, were obtained from the official echocardiographic report. LV mass indexed to the body surface area was estimated by the LV cavity dimension and wall thickness at the end-diastolic phase. Valve regurgitation was classified as grade 0 (none), 1 (trace), 2 (mild), 3 (moderate), or 4 (severe). The serum brain natriuretic peptide (BNP) level was measured at 1, 6, and 12 months after LVAD implantation. The patients were stratified by improvement of LVEF at 6 months post-LVAD implantation: ≥ 10% increase (Gr ≥ 10%), 5–10% (Gr 5–10%), and ≤ 5% (Gr ≤ 5%).

### Pathohistological analyses

Sampled LV apex tissue at the time of LVAD implantation surgery was fixed in formalin and embedded in paraffin. Five-micrometre-thick sections were stained with haematoxylin/eosin or Masson’s trichrome to analyse the cardiomyocyte size or collagen area, respectively. Additionally, the sections were immunolabelled with mouse monoclonal antibodies against CD3 (1/10 dilution; N1580 clone, Dako, Agilent Technologies, Santa Clara, CA, USA) or CD68 (1/1000 dilution; M0814 clone, Dako) to label T cells or macrophages, respectively. The immunolabelled sections were visualized by a Bond Polymer Refine Detection Kit (DS9800; Leica Biosystems, Wetzlar, Germany).

Whole section images were scanned with an Aperio Scan-Scope XT (Leica Biosystems) and digital sections were viewed using Aperio ImageScope software (Leica Biosystems). Photomicrographs were recorded in five randomly selected high power fields and a low magnification whole view of the digitised image. The size of cardiomyocytes was evaluated in sections stained with haematoxylin/eosin by measurement of ≥ 30 cardiomyocytes in high power fields. The collagen area fraction in the whole myocardial area was determined automatically by Aperio ImageScope software. The numbers of infiltrating CD3- and CD68-positive cells in photomicrographs were counted in a blinded manner.

### Statistical analysis

Statistical analysis was performed using JMP^®^ 13 (SAS Institute Inc., Cary, NC, USA). All quantitative data are expressed as the mean ± standard deviation (SD). The statistical significance of differences was analysed using the unpaired Student’s *t* test for parametric continuous variables and the Mann–Whitney *U* test for non-parametric continuous variables. Event-free survival curves were constructed using the Kaplan–Meier method.

## Results

### Background and characteristics

The backgrounds and characteristics of the patients, which included 50 cases with NIDCM, are presented in Table 1. The duration of heart failure was defined as the interval between diagnosis and/or the start of medical treatment for heart failure and LVAD implantation. Most patients had received optimal medical treatment by β-blockers, angiotensin-converting enzyme inhibitors, and/or angiotensin receptor blockers for an average duration until surgery of 80 months. Prior cardiac surgery had been performed in 11 patients (22%), such as extracorporeal LVAD implantation. Most patients were categorised as INTERMACS Profile 3 at the time of LVAD implantation.

**Table 1:** Patient characteristics and baseline

Characteristics	*n* = 50
Age, years	42 ± 12
Male, *n* (%)	41(82)
BMI, kg/m^2^	21.7 ± 4.9
BSA, m^2^	1.68 ± 0.20
Duration of heart failure, months	82 ± 87
NYHA class	
Class III, *n* (%)	15(30)
Class IV, *n* (%)	35(70)
INTERMACS profile	
Profile 2, *n* (%)	11(22)
Profile 3, *n* (%)	32(64)
Profile 4, *n* (%)	1(2)
Bridge to bridge, *n* (%)	6(12)
Hypertention, *n* (%)	2(4)
Dyslipidemia, *n* (%)	17(34)
Diabetes, *n* (%)	10(20)
ICD or CRT-D, *n* (%)	27(54)
Preoperative thoracotomy, *n* (%)	11(22)
IABP, *n* (%)	7(14)
CRRT, *n* (%)	0(0)

Blood test	*n* = 50
Hemoglobin, g/dl	11.7 ± 2.2
Total bilirubin, mg/dl	1.1 ± 0.9
Creatinine, mg/dl	0.9 ± 0.3
CRP, mg/dl	1.6 ± 2.5
BNP, pg/ml	683 ± 660

Echocardiography	*n* = 50
LVDD, mm	75 ± 11
LVSD, mm	69 ± 12
LVEF, %	17 ± 6
LAD, mm	44 ± 12
LV mass index, g/m^2^	148 ± 50
AR ≧ moderate, *n* (%)	0(0)
MR ≧ moderate, *n* (%)	19(38)
TR ≧ moderate, *n* (%)	1(2)

Haemodynamics	*n* = 47
Heart rate, bpm	83 ± 20
Systolic blood pressure, mmHg	89 ± 13
PCWP, mmHg	20 ± 11
mean PAP, mmHg	29 ± 13
Cardiac index, l/min/m^2^	2.0 ± 0.7
Forrester type	
Type 1, *n* (%)	9(19)
Type 2, *n* (%)	3(6)
Type 3, *n* (%)	11(23)
Type 4, *n* (%)	22(47)
RAP/PCWP > 0.5, *n* (%)	11(23)

Medication	*n* = 50
β-blocker, *n* (%)	45(90)
Dose of β-blocker^a^, mg	12 ± 7
ACE inhibitor or ARB, *n* (%)	40(80)
MRA, *n* (%)	42(84)

*BMI* body mass index, *BSA* body surface area, *NYHA* New York Heart Association, *INTERMACS* Interagency Registry for Mechanically Assisted Circulatory Support, *ICD* implantable cardioverter defibrillator, *CRT-D* cardiac resynchronization therapy defibrillator, *IABP* intra-aortic balloon pumping, *CRRT* continuous renal replacement therapy, *CRP* C-reactive protein, *BNP* brain natriuretic peptide, *LVDD* left ventricular end-diastolic dimension, *LVSD* left ventricular end-systolic dimension, *LVEF* left ventricular ejection fraction, *LAD* left atrial dimension, *LV* left ventricular, AR aortic regurgitation, *MR* mitral regurgitation, *TR* tricuspid regurgitation, *PCWP* pulmonary capillary wedge pressure, *PAP* pulmonary artery pressure, *RAP* right atrial pressure, *ACE* angiotensin-converting enzyme, *ARB* angiotensin II receptor blocker, *MRA* mineralocorticoid receptor antagonist

^a^The presented doses were converted to the carvedilol equivalent

Echocardiographically, all patients showed substantial dilatation of the LV and poor LVEF without regional wall motion abnormalities. Moderate tricuspid regurgitation was noted in one patient, whereas no cases showed moderate or more aortic regurgitation. Right heart catheter analysis showed that 26 and 11 patients were categorized as Forrester Type 4 and Type 3, respectively. The RV function was estimated by a right atrial pressure/pulmonary capillary wedge pressure ratio of > 0.5, so that 11 patients were categorized as right ventricular (RV) failure. Thirteen patients (26%) showed serum BNP of > 800 pg/ml. All patients were histologically diagnosed as NIDCM by a biopsy specimen of the RV and/or a resection specimen of the LV apex prior to LVAD implantation.

### Operative procedures, and in-hospital and mid-term outcomes

LVAD was implanted via the median sternotomy approach. Tricuspid annuloplasty was performed in 11 patients (22%) who had mild or more tricuspid regurgitation. Aortic valve plasty by a coaptation stitch [13] was conducted in three patients (6%) of the cases with mild aortic regurgitation. Right VAD was added in one patient (Table 2).

**Table 2:** Operative procedures

Operative procedures	*n* = 50
Operation time, min	295 ± 111
Cardiopulmonary bypass time, min	94 ± 34
TAP, *n* (%)	11 (22)
AVP, *n* (%)	3 (6)
AVR, *n* (%)	0 (0)
RVAD, *n* (%)	1 (1)

In-hospital outcome	*n* = 50
In-hospital mortality, *n* (%)	1 (2)
ICU stay, days	5 ± 4
RV failure, *n* (%)	1 (2)

*TAP* tricuspid annuloplasty, *AVP* aortic valvuloplasty, *AVR* aortic valve replacement, *RVAD* right ventricular assist device, *ICU* intensive care unit, *RV* right ventricular

There was one in-hospital mortality because of cerebral infarction (2%). One patient was categorized as RV failure by prolonged inotrope support post-operatively. No patient was tracheotomised. Intensive care unit stay was an average of 5 days. All surviving patients were discharged without permanent disorders such as neurological deficit or maintenance haemodialysis.

After discharge, there was one mortality until the latest follow-up because of cerebral infarction at 38 months post-LVAD implantation. Four patients (8%) presented with congestive cardiac failure that was treated in-hospital. Among them, two patients had received multiple in-hospital treatments for congestive cardiac failure. Congestive cardiac failure was caused by aortic valve regurgitation and/or RV failure. Other complications after discharge included driveline infection in 13 patients, gastrointestinal bleeding in 4, and cerebral infarction in 5. Kaplan–Meier analysis revealed that 1- and 5-year survival rates were 98 and 92%, respectively and that 1- and 5-year avoidance rates of congestive cardiac failure treated in-hospital were 96 and 89%, respectively.

### LV functional recovery post-LVAD implantation

**Fig. 1:**
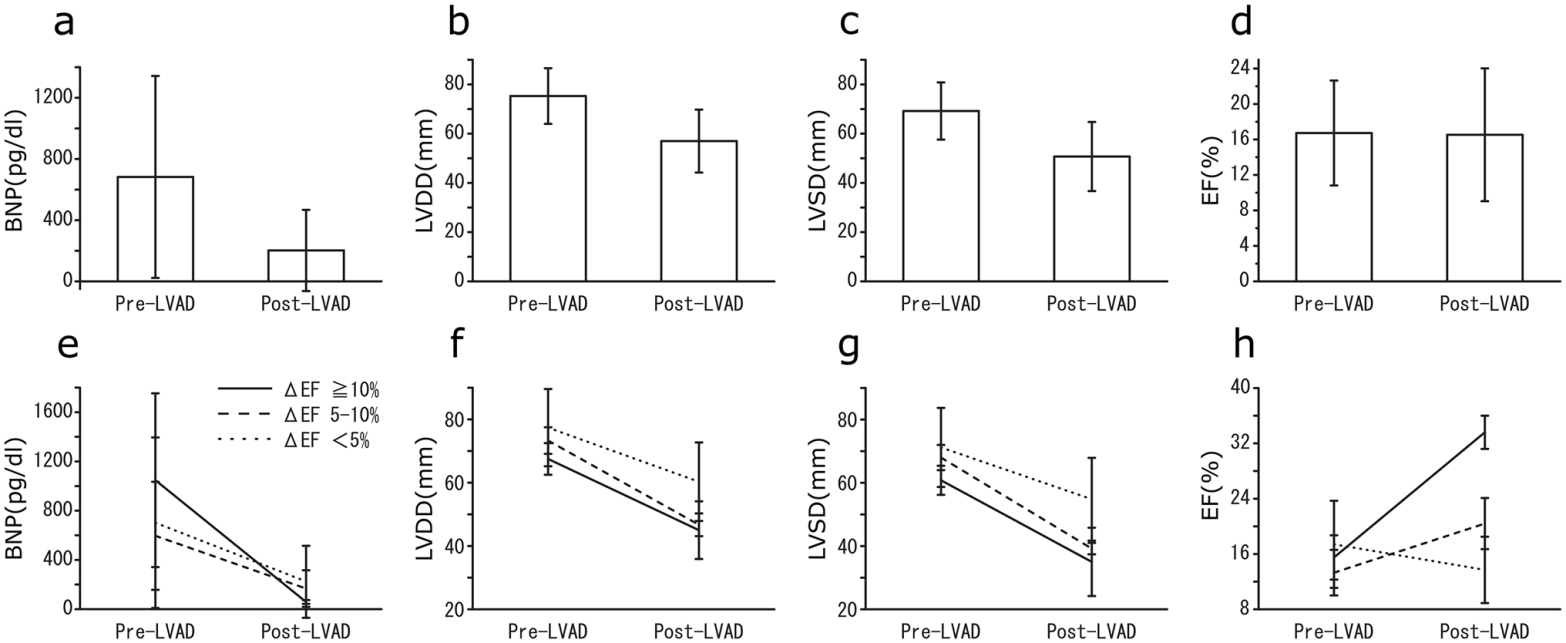
Echocardiographic measurements and serum brain natriuretic peptide (BNP) before and at 6 months after left ventricular assist device (LVAD) implantation. **e**–**h** Echocardiographic measurements and BNP are shown for each group on the basis of changes in the left ventricular ejection fraction (LVEF)

All in-hospital surviving patients (n = 49) were examined by standard transthoracic echocardiography within 7 days prior to LVAD implantation and at an average of 6.0 months (5.0–6.3 months) post-LVAD implantation. As a result, the LVEF was constant post-LVAD implantation, while LVDD and LVSD were decreased significantly post-LVAD implantation (Fig. 1). Conversely, serum BNP was significantly reduced post-LVAD implantation.

The patients were stratified by improvement of LVEF at 6 months post-LVAD implantation: Gr ≥ 10%; *n* = 4, Gr 5–10%; *n* = 6, and Gr ≤ 5%: *n* = 39. LVDD and LVSD in patients with Gr ≥ 10% were reduced significantly post-LVAD implantation, whereas those in patients with Gr ≤ 5% were not significantly different between pre- and post-LVAD implantation. The reduction rate in serum BNP post-LVAD implantation in patients with Gr ≥ 10% was significantly greater than that in other groups.

By comparing clinical characteristics in each group, the durations of heart failure were 88 ± 89 months, 79 ± 66 months, and 48 ± 61 months in patients with Gr ≤ 5%, Gr 5–10%, and Gr ≥ 10%, respectively. The numbers of patients with an implantable cardioverter defibrillator (ICD) or cardiac resynchronization therapy defibrillator (CRT-D) were 19 (49%), 0 (0%), and 2 (50%) in patients with Gr ≤ 5%, Gr 5–10%, and Gr ≥ 10%, respectively. The numbers of patients with moderate or severe mitral regurgitation were 22 (52%), 3 (6%), and 2 (50%) in patients with Gr ≤ 5%, Gr 5–10%, and Gr ≥ 10%, respectively. The QRS intervals were 121 ± 25 ms, 101 ± 14 ms, and 140 ± 45 ms in patients with Gr ≤ 5%, Gr 5–10%, and Gr ≥ 10%, respectively. The β-blocker doses were 12 ± 7 mg, 12 ± 7 mg, and 10 ± 2 mg in patients with Gr ≤ 5%, Gr 5–10%, and Gr ≥ 10%, respectively.

### Presence of immunocompetent cells in the myocardium

**Fig. 2:**
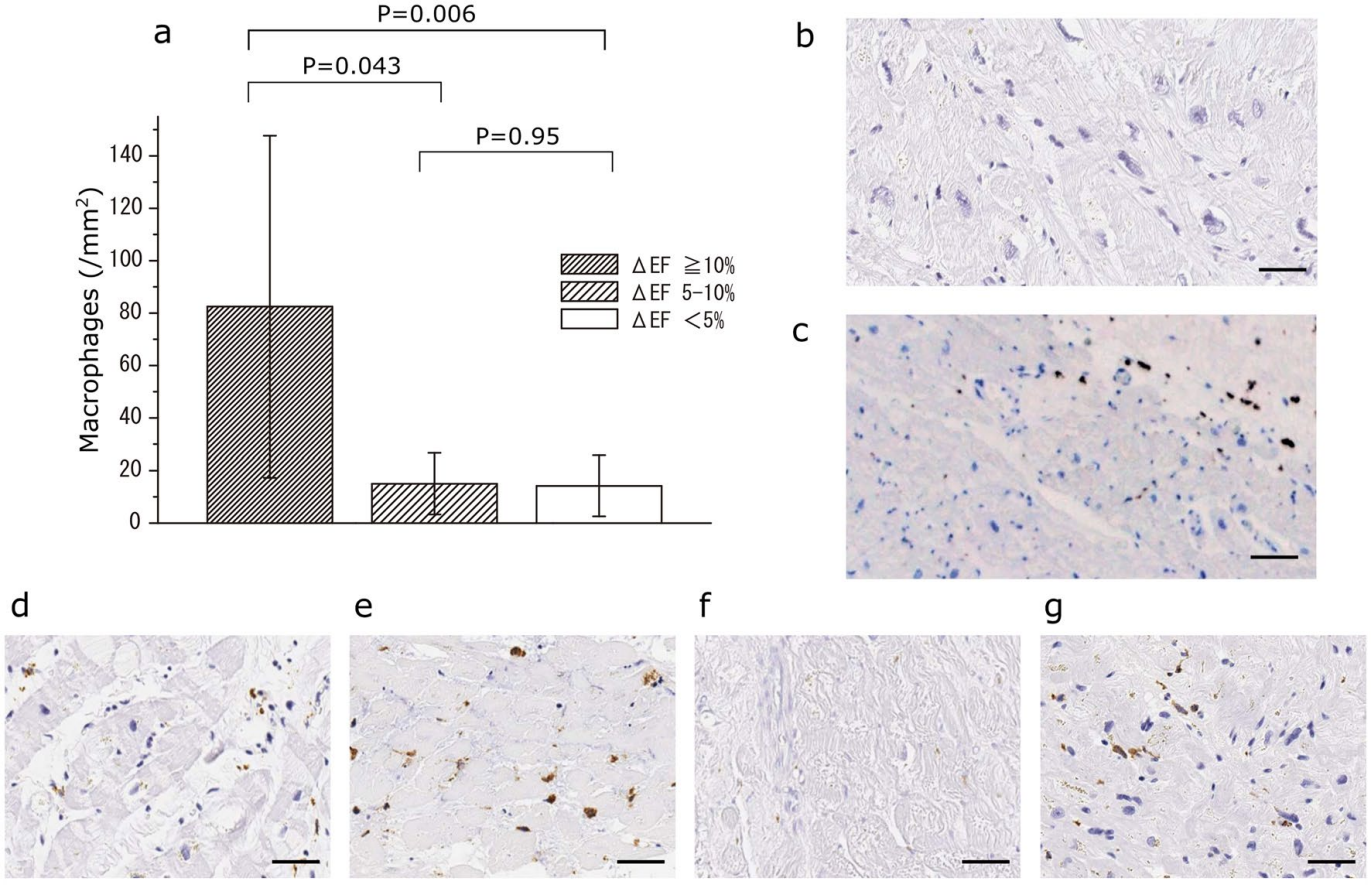
**a** Mean numbers of macrophages (± SD) are shown for each group on the basis of changes in the left ventricular ejection fraction (LVEF). **b** Representative immunohistochemical staining of a smaller number of macrophages in a myocardium specimen in a patient with Gr ≤ 5%. **c** Representative immunohistochemical staining of a greater number of macrophages in a myocardium specimen in a patient with Gr ≤ 5%. **d**–**g** Immunohistochemical staining of myocardium specimens in each patient with Gr ≥ 10%. Macrophages positive for CD68 are stained brown. Scale bar: 40 µm

The number of CD68-positive macrophages in the myocardium of patients with Gr ≥ 10% (83 ± 74/mm^2^) was significantly greater than that of patients with Gr 5%–10% and Gr ≤ 5% (15 ± 12/mm^2^ and 14 ± 11/mm^2^, respectively) (Fig. 2). Among patients with Gr ≥ 10%, all four cases showed a homogeneous distribution of CD68-positive macrophages (Fig. 2).

**Fig. 3:**
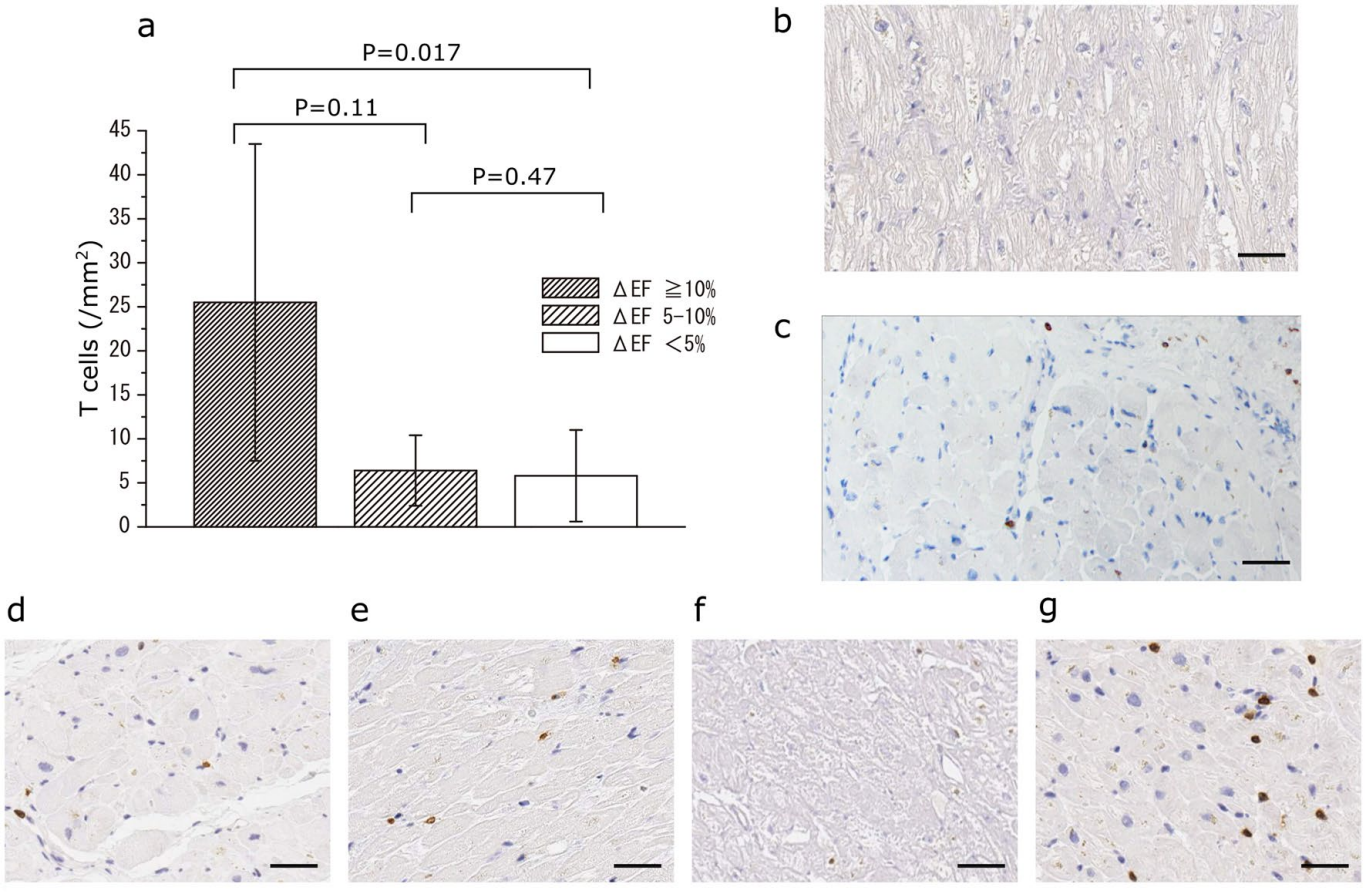
**a** Mean numbers of T cells (± SD) are shown for each group on the basis of changes in the left ventricular ejection fraction (LVEF). **b** Representative immunohistochemical staining of a smaller number of T cells in a myocardium specimen in a patient with Gr ≤ 5%. **c** Representative immunohistochemical staining of a greater number of T cells in a myocardium specimen in a patient with Gr ≤ 5%. **d**–**g** Immunohistochemical staining of myocardium specimens in each patient with Gr ≥ 10%. T cells positive for CD3 are stained brown. Scale bar: 40 µm

The number of CD3-positive T cells in the LV myocardium was significantly smaller than that of CD68-positive macrophages in all groups. Among the three groups, the number of CD3-positive T cells in the myocardium of patients with Gr ≥ 10% (26 ± 20 /mm^2^) was significantly greater than that of patients with Gr 5–10% and Gr ≤ 5% (6 ± 4/mm^2^ and 5 ± 5/mm^2^, respectively) (Fig. 3). Among patients with Gr ≥ 10%, all four cases showed a homogeneous distribution of CD3-positive T cells (Fig. 3).

### Histological reverse remodelling post-LVAD implantation

**Fig. 4:**
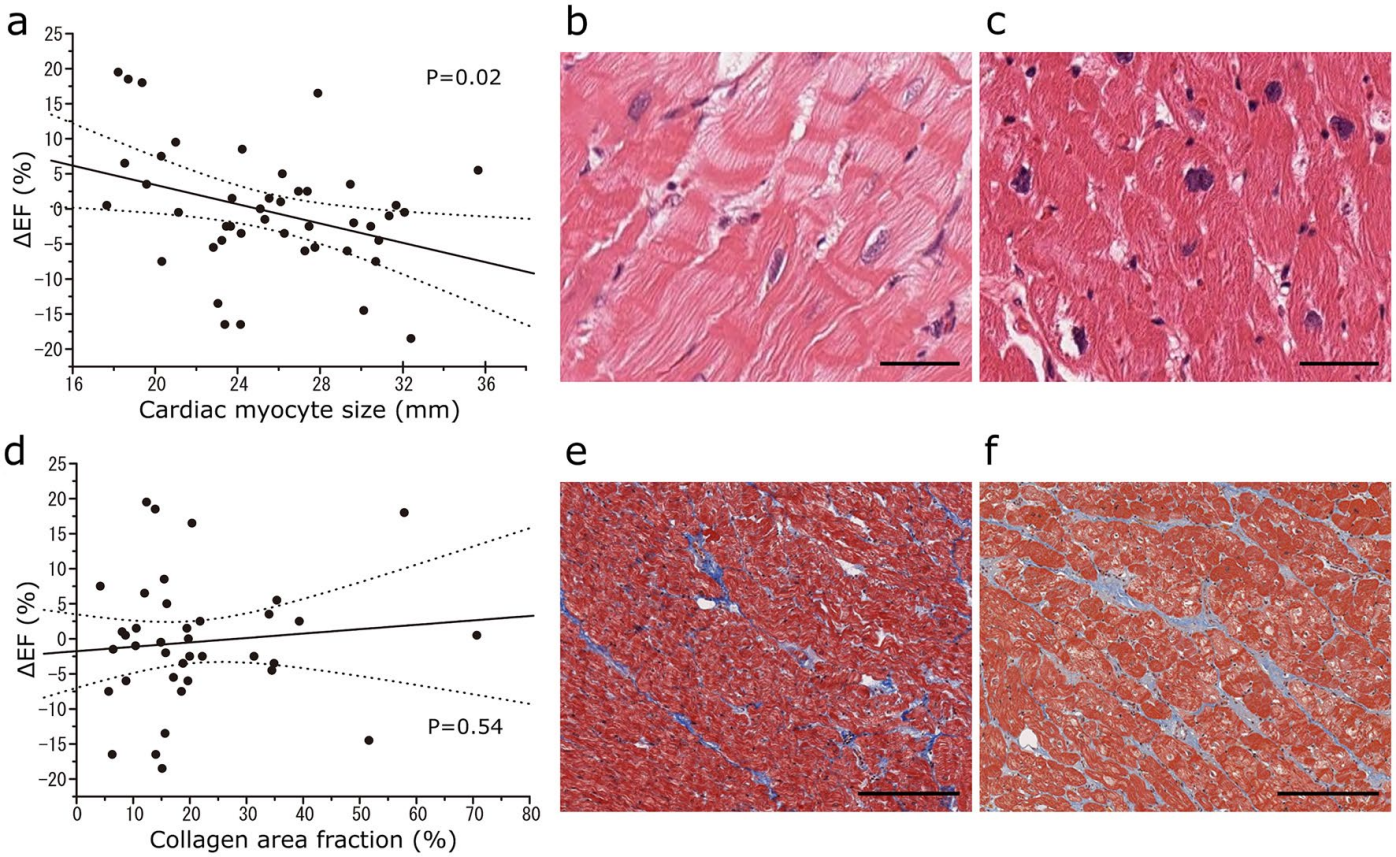
**a** Correlation between the cardiomyocyte size at the time of left ventricular assist device (LVAD) implantation and the change in the left ventricular ejection fraction (LVEF) between preimplantation and at 6 months after implantation. The dotted line shows the 95% confidence interval (CI). **b** Representative haematoxylin–eosin (HE) staining of a myocardium specimen in the group with LVEF improvement of < 5%. Scale bar: 20 µm. **c** Representative HE staining of a myocardium specimen in the group with LVEF improvement of ≥ 10%. Scale bar: 20 µm. **d** Correlation between the collagen area fraction at the time of LVAD implantation and change in LVEF between preimplantation and at 6 months after implantation. The dotted line shows the 95% CI. **e** Representative Masson’s trichrome (M-T) staining of a myocardium specimen in the group with LVEF improvement of < 5%. Scale bar: 400 µm. **f** Representative M-T staining of a myocardium specimen in the group with LVEF improvement of ≥ 10%. Scale bar: 400 µm

Relationships between the change in LVEF and the myocyte size or collagen area were analysed statistically. There was a significant negative correlation between the increase of LVEF and the myocyte size (Fig. 4a). Conversely, there was no significant correlation between the increase of LVEF and the collagen area (Fig. 4d). Relationships between the number of CD68-positive macrophages or CD3-positive T cells in the myocardium and the myocyte size or collagen area were also analysed statistically. There was no significant correlation between the number of CD68-positive macrophages (*p* = 0.22) or CD3-positive T cells (*p* = 0.086) and myocyte size. Similarly, there was no significant correlation between the number of CD68-positive macrophages (*p* = 0.063) or CD3-positive T cells (*p* = 0.18) and the collagen area.

## Discussion

Out of 50 patients, 4 with NIDCM-induced advanced cardiac failure showed substantial cardiac functional recovery after LVAD implantation. LVEF recovery was associated with reductions in the LV size and serum BNP. Additionally, greater numbers of CD68-positive macrophages and CD3-positive T cells were present in patients with functional recovery, which indicated that the viability of cardiac tissue may be, at least in part, determined by immunocompetent cells and myocardial inflammation. There were no significant differences in accumulation of immunocompetent cells between Gr 5–10% and Gr ≤ 5%. In contrast, cut-off value of LVEF recovery at 6-monthes post-LVAD implantation reportedly 5% in some publications [14]. In addition, sample size of the Gr = > 10% was 4 cases in the present work. It might that the Gr 5–10% represent cases having significant reverse remodelling with some contribution.

Nakayama et al. reported that the presence of macrophages and T cells was associated with poor outcomes of NIDCM patients [15]. These results appear to be inconsistent with those of the present study in which the presence of macrophages and T cells was associated with reverse LV remodelling in NIDCM patients. This inconsistency can be explained by the difference in the pathological stages of NIDCM. While the cohort of the previous study was categorized as stage B or C heart failure, that of the present study was categorized as stage D heart failure. Pathological progression of NIDCM is associated with various factors such as mitral regurgitation, blood pressure, medical treatments, physical activities, and involvement of myocardial inflammation. In stage B and C heart failure, myocardial inflammation, and particularly M2-like macrophage activity, which directly correlate with fibrotic activity, indicate progressive myocardial tissue damage. Conversely, the presence of these cells during stage D heart failure may indicate that the remaining viable myocardial tissue is under progressive damage. It has been reported that some molecular changes occur during LVAD support, such as normalisation of disrupted Ca^2+^ transport, reductions in cytokines, improvements in heart failure-related gene expression, upregulation of β-adrenergic receptors, and reductions in β1-adrenoceptor autoantibodies [16, 17]. These effects might be more likely to occur in a heart with more viable remaining myocardium. In this study, the area of fibrosis in the myocardium was not related to the degree of functional reverse remodelling at the time of LVAD implantation. Conversely, the size of myocytes was significantly related to functional reverse remodelling. We consider that LV unloading by LVAD implantation would have activated CD3- and CD68-positive cells in the heart, which contributed to the reparative process that would induce reverse remodelling, including a reduction in fibrosis. Conversely, a large myocyte size would indicate more diseased myocytes. Furthermore, functional reverse remodelling was related to a short duration of heart failure. The absence of immunocompetent cells during stage D heart failure might indicate completion of the myocardial damage process and poor viability of these tissues due to the progression of the pathological stage.

Preoperative ICD, CRT-D, or a β-blocker might affect the remodelling process of the LV after LVAD implantation. However, there were no statistically significant relationships between the number of patients with ICD/CRT-D or the β-blocker dose and the degree of functional recovery in this study. This may be explained by the fact that all cases were treated by optimized device/medical therapies prior to the LVAD implantation. Furthermore, there are reportedly multiple predictive factors of reverse remodelling in NIDCM, such as the duration of heart failure, the degree of mitral regurgitation (MR), and QRS interval. There was a significant correlation between the degree of functional recovery and the duration of heart failure. Conversely, MR or the QRS interval at the time of LVAD implantation did not show a significant correlation with the degree of functional recovery. NIDCM is a progressive disease represented by gradual development of myocardial tissue damage. The duration of heart failure is therefore an important factor to assess myocardial viability. LV unloading by LVAD implantation would activate the reverse remodelling process more effectively in more viable myocardium. Conversely, MR and a wide QRS would reflect heterogenous or end-stage myocardial damage, either of which would be less affected by LV unloading.

The results of the present study have important implications in contemporary clinical practice for patients with stage D heart failure as follows. First, the presence of immunocompetent cells in the LV apex at the time of LVAD implantation surgery predicted long-term outcomes under LVAD support. Intensive medical treatments or early surgical intervention, which include transplantation of a marginal donor heart, might improve outcomes of patients with poorly distributed immunocompetent cells. Second, patients who have abundant immunocompetent cells might be good candidates for LVAD weaning. Additional treatments, such as cardiac regeneration therapy, might be indicated for bridge-to-recovery in this patient population.

This study is limited by the small number of enrolled patients with only four cases showing substantial functional recovery post-LVAD implantation. Despite the relatively consistent results in this study, a larger number of enrolled patients would confirm the findings of this study and contribute to more detailed data, particularly histological results. Additionally, the roles of CD3-positive T cells and/or CD68-positive macrophages were uncertain in this study. Therefore, more specific immunohistological assessments and experimental animal studies are warranted.

## Conclusion

In conclusion, despite aggressive medical treatments post-LVAD implantation, functional recovery was not frequently seen in advanced NIDCM patients. Immunocompetent cells in the LV myocardium might be important to predict myocardial viability of this pathology.
